# Aortic valve timing is critical for accurate estimation of MRI-derived ejection fraction

**DOI:** 10.1186/1532-429X-16-S1-P327

**Published:** 2014-01-16

**Authors:** Francisco Contijoch, Kelly Rogers, Walter R Witschey, Robert C Gorman, Yuchi Han

**Affiliations:** 1 University of Pennyslvania, Philadelphia, Pennsylvania, USA

## Background

We aim to investigate the effect of utilizing aortic valve timing in the measurement of cardiac magnetic resonance (CMR)-derived ejection fraction (EF). Although CMR-derived left ventricular (LV) EF is the gold-standard for volumetric evaluation of the heart, it always reports higher values when compared to other modalities such as echocardiography and radionuclide ventriculography. The higher EF has often been attributed to better endocardial definition by CMR. In the ACCF/ACR/AHA/NASCI/SCMR 2010 Expert Consensus Document on CMR, a 1987 paper is cited with utilizes the "maximal and minimal left ventricular cross-sectional area at the mid-ventricle" to identify end-systole and end-diastole. The 2013 CMR Guideline suggests phases with the largest and smallest global LV blood volume be utilized to identify end-diastole and end-systole. The same guideline also suggests aortic valve timing be utilized when arrhythmia or mitral regurgitation is encountered. We investigated the effect of different schemes on measured LVEF.

## Methods

Standard retrospectively EKG-gated bSSFP short axis and LVOT cine images in 46 clinical patients in normal sinus rhythm with a range of EF (5.8 - 75.8 %) were acquired and analyzed. Scan parameters were as follows: in-plane resolution: 1.25 - 2.08 mm, slice spacing: 8 mm with a gap of 2 mm, reconstructed temporal resolution: 18.2 - 58.8 ms. Aortic valve opening and closing as a percentage of cardiac cycle was identified on LVOT images. Volumetric evaluation of short axis images was performed via semi-automated segmentation for all phases and all left ventricular slices (ITK-SNAP, Philadelphia PA). The schemes (shown in Table 1) were used to quantify LVEF. Scheme G was considered "true EF" and usied aortic valve opening and closing. Scheme F is intended to mimic real-time methods, which acquire slice-by-slice data and do not incorporate aortic valve timing or other external measures such as EKG.

**Table 1:** 

Scheme	End-Diastolic Phase	End-Systolic Phase	EF Error (EF = 35 %)	EF Error (EF = 70 %)
A	1st Phase after EKG Trigger	Phase when mid-ventricular slice area reaches minimum.	3.1%	4.6%

B	2nd Phase after EKG Trigger		2.3%	3.5%

C	1st Phase before EKG Trigger		3.0%	4.4%

D	2nd Phase before EKG Trigger		3.4%	5.1%

E	Phase of Max Global Volume	Phase of Min Global Volume	4.7%	6.4%

F	Phase of Max Slice Volume	Phase of Min Slice Volume	7.0%	8.7%

G	Aortic Valve Opening	Aortic Valve Closing	--	--

## Results

**Figure 1:**
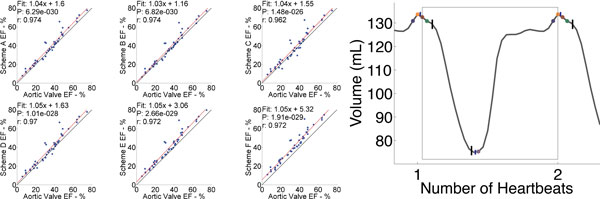
**LEFT The correlation plots for each of the schemes outlines in the table and Aortic Valve based ejection fraction are shown on the left side of the figure**. For all schemes, the error in measured EF increases with increasing EF (slope > 1). The table illustrates expected error at tow different ejection fractions. RIGHT: Global volume over time curve for a patient with an EF = 45% is shown. The red, green, blue and orange dots represent phases around the EKG trigger which correspond to Schemes A-D. The mid-ventricular end-systole is denoted by a purple dot. The aortic valve opening and closure are shown by two horizontal black bars. The horizontal blue lines indicate the average phases over which individual slices obtain their local maxima or minima.

Figure 1 illustrates the results of the different schemes. Linear fits were calculated between each proposed methods and the aortic valve based measure. The right side of the Figure illustrates the different phases selected using different schemes. In most patients, aortic valve opening occurs after a small decrease in global volume and the aortic valve closes prior to reaching the global minimum value. Table 1 illustrates that the clinical methods overestimate EF in a linear (EF-dependent) manner ranging from 3.1 to 8.7 %.

## Conclusions

Aortic valve timing results in a small but consistent decrease in ejection fraction relative to current standard techniques. Furthermore, if real-time techniques which acquire slice-by-slice data are not synchronized with external timing information such as aortic valve timing or EKG, the stroke volume will be overestimated leading to an additional overestimation of EF.

## Funding

K99-HL108157, R01-HL103723, T32HL007954, T32-EB009384.

